# Under recognition and treatment of lymphedema in head and neck cancer survivors – a database study

**DOI:** 10.1007/s00520-023-07698-3

**Published:** 2023-03-23

**Authors:** Michael D. Stubblefield, Derek Weycker

**Affiliations:** 1 Kessler Institute for Rehabilitation, 1199 Pleasant Valley Way, West Orange, NJ 07052 USA; 2 Policy Analysis Inc. (PAI), 822 Boylston Street, Suite 206, Chestnut Hill, MA USA

**Keywords:** Head and neck cancer, Lymphedema, Rehabilitation, Function, Quality of life, Physical therapy, Fibrosis

## Abstract

**Purpose:**

Head and neck cancer (HNC) will be diagnosed in approximately 54,000 Americans in 2022 with more than 11,000 dying as a result. The treatment of HNC often involves aggressive multimodal therapy including surgery, radiotherapy, and systemic therapy. HNC and its treatments are associated with multiple painful and function-limiting neuromusculoskeletal and visceral long-term and late effects. Among these is head and neck lymphedema (HNL), the abnormal accumulation of protein rich fluid, in as many as 90% of survivors. Though HNL is common and potentially contributory to other function-limiting issues in this population, it is notoriously understudied, underrecognized, underdiagnosed, and undertreated. This study seeks to determine the incidence of HNC-related lymphedema diagnosis and treatment in a large US healthcare claims repository database.

**Methods:**

A retrospective observational cohort design and data from an integrated US healthcare claims repository—the IBM MarketScan Commercial Claims and Encounters (CCAE) and Medicare Supplemental and Coordination of Benefits (MDCR) Databases spanning the period April 1, 2012 through March 31, 2020.

**Results:**

Of the 16,654 HNC patients eligible for evaluation, 1,082 (6.5%) with a diagnosis of lymphedema were identified based on eligibility criteria. Of the 521 HNC patients evaluated for lymphedema treatment, 417 (80.0%) patients received 1.5 courses of MLD, 71 (13.6%) patients were prescribed compression garments, and 45 (8.6%) patients received an advanced pneumatic compression device.

**Conclusion:**

HNL in this population of HNC survivors was underdiagnosed and treated compared with contemporary assessments HNL incidence.

## Introduction

The American Cancer Society estimates that 54,000 Americans will develop head and neck cancer (HNC) in 2022 and more than 11,000 will die as a result [1]. Aggressive, multi-modal therapy is the mainstay of treatment for patients with locally advanced disease. Such treatments, though potentially curative, often come at the price of severe, painful, function- and quality of life-limiting acute and late effects [2]. The epidemic of human papilloma virus (HPV)-associated HNC, a disease that is generally more curable, but also most prevalent in younger individuals, has shifted the burden of HNC-related impairments to a younger cohort [3]. Improved treatment outcomes coupled with this changing epidemiology will result in many more HNC survivors destined to live their lives, often from a relatively young age, with the late effects of HNC and its treatment [4, 5].

Lymphedema is characterized by several pathophysiological events, including lymph stasis, lymphatic vessel remodeling and dysfunction, inflammation, adipose tissue deposition, and ultimately fibrosis [6]. Head and neck lymphedema (HNL), a common complication of HNC, results from treatments such as neck dissection and/or radiation. HNL can progress and cause chronic inflammatory, fibrosclerotic and fibrofatty deposition resulting in permanent deformity and disability [7, 8]. The reported prevalence of lymphedema in HNC survivors ranges from 12% to more than 90% [8–12]. This variation is due to methodological differences between studies with contemporary studies favoring the high range of prevalence. [8, 12]

HNL is not simply a cosmetic issue. It is both an external and internal phenomenon with significant clinical sequelae [13, 14]. Ongoing fibrosis and inflammation associated with HNL are likely to contribute to progression of associated late effects [5, 8, 15]. Functional sequelae associated with HNL include skin changes, pain, range of motion limitations, contracture, dysphagia, dysarthria, dyspnea, postural abnormalities, trismus, and reduced quality of life among other issues [5, 8, 14, 16–19]. Progression of HNL and fibrosis is associated with increasing symptom burden, functional impairment, and reduced quality of life [5, 17]. There is growing evidence in the breast cancer literature that early recognition and effective treatment of breast cancer-related lymphedema improves outcomes [20, 21]. Though less robust, evidence also suggests that the early identification and effective treatment of HNL may improve functional outcomes including dyspnea, pain, and dysphagia in HNC survivors [22, 23].

Despite a prevalence of more than 90% in HNC survivors and the potential benefits of early and effective treatment, HNL is understudied, underrecognized, underdiagnosed, and undertreated [8, 9, 13]. The magnitude of this deficiency has not been well defined. This study seeks to determine the incidence of HNC-related lymphedema diagnosis and treatment in a large US healthcare claims repository database.

## Methods

### Study design and data source

A retrospective observational cohort design and data from an integrated US healthcare claims repository—the IBM MarketScan Commercial Claims and Encounters (CCAE) and Medicare Supplemental and Coordination of Benefits (MDCR) Databases—were employed. For this study, data spanned the period April 1, 2012 through March 31, 2020.

The CCAE Database includes healthcare claims and enrollment information from employer-sponsored plans throughout the US that provide health benefits to working persons aged < 65 years annually, including the employees, their spouses, and their dependents. The MDCR Database includes healthcare claims and enrollment information for retirees who are Medicare-eligible and have elected to enroll in employer-sponsored Medicare supplemental plans (and for which both the Medicare-paid amounts and employer-paid amounts are available).

Healthcare claims include medical (i.e., facility and professional service) and outpatient pharmacy claims. Data available for each facility and professional service claim include the dates and places of service, diagnoses, procedures performed/services rendered, and quantity of services (professional-service claims). Data available for each outpatient pharmacy claim include the drug dispensed, dispensing date, dose, quantity dispensed, and number of therapy days supplied. Medical and pharmacy claims also include amounts paid (i.e., reimbursed) by health plans and patients to healthcare providers for services rendered. Selected demographic and eligibility information also is available. Patient-level data can be arrayed chronologically to provide a detailed longitudinal profile of all medical and pharmacy services used by each plan member.

### Study population

Two populations of patients were identified from this dataset – those with HNC-related lymphedema and those who received treatment for HNC-related lymphedema.

### HNC incidence

For analyses of HNC incidence, the study population comprised patients aged ≥ 18 years who had first evidence of HNC between April 1, 2013 and March 31, 2019, and who received treatment for this condition during the 3-month period following the initial diagnosis. Evidence of HNC was ascertained based on ≥ 1 diagnosis code in the acute-care hospital (inpatient) setting, or ≥ 2 diagnosis codes—at least seven days apart—in the ambulatory (outpatient) setting; evidence of HNC treatment was ascertained based on corresponding procedure codes (Appendix). Patients were excluded from the study population if they were not continuously enrolled during the 1-year period preceding, and during the 1-year period following, first evidence of HNC, or if they had evidence of lymphedema or other etiologies for lymphedema (e.g., other cancers, venous leg ulcers, chronic venous insufficiency) prior to the initial HNC diagnosis.

### HNC-related lymphedema incidence

For analyses of HNC-related lymphedema incidence, the study population comprised patients aged ≥ 18 years who had first evidence of lymphedema between April 1, 2013 and March 31, 2019, and who had evidence of HNC—as well as cancer-related treatment—during the 1-year period preceding the initial diagnosis of lymphedema. Evidence of lymphedema (primary/secondary) was ascertained based on ≥ 1 diagnosis code for lymphedema in the acute-care hospital (inpatient) setting, or ≥ 2 diagnosis codes for lymphedema—at least seven days apart—in the ambulatory (outpatient) setting (Appendix). Patients were excluded from the study population if they were not continuously enrolled during the 1-year period preceding, and during the 1-year period following, first evidence of lymphedema, or if they had evidence of other etiologies for lymphedema (e.g., other cancers, venous leg ulcers, chronic venous insufficiency).

### Baseline characteristics

Baseline characteristics of lymphedema patients with head/neck cancer were ascertained during the 1-year period prior to the initial diagnosis of lymphedema, and included: demographic profile (age, sex); clinical profile (lymphedema-related conditions, comorbidities); and cancer treatment profile (chemotherapy, radiation, surgery). Lymphedema-related conditions and comorbidities were identified based on encounters with a corresponding diagnosis code. Operational algorithms and codes used to ascertain baseline characteristics are set forth in the Appendix.

### HNC-related lymphedema treatment incidence

Use of lymphedema treatment among HNC patients was ascertained during the 1-year period following first evidence of lymphedema. Treatments included conservative (CONS) therapy (manual lymphatic drainage [MLD], compression garments [CG]) and advanced pneumatic compressions devices (APCD).

Each unique treatment course was identified, beginning with the first, and all qualifying encounters (i.e., with the same Current Procedural Terminology [CPT] or Healthcare Common Procedure Coding System [HCPCS] code) occurring within 30 days of each other was deemed to be part of the same treatment course.

## Results

Patient demographics and baseline characteristics are detailed in Table 1.

**Table 1:** Demographics and baseline characteristics

	HNC + LED patients (*N* = 521)
Age (years)	
Mean (SD)	58.0 ± 9.6
Groups	
18–34	8 (1.5%)
35–44	27 (5.2%)
45–54	131 (25.1%)
55–64	275 (52.8%)
65–74	48 (9.2%)
≥ 75	32 (6.1%)
Sex	
Female	12 (23.2%)
Male	509 (76.8%)
LED-Associated Conditions	
Iliac vein disorders (May-Thurner syndrome)	10 (0.2%)
Cellulitis	17 (3.3%)
Deep vein thrombosis	24 (4.6%)
Other infections	287 (55.1%)
Comorbidity Profile	
Depression	162 (31.1%)
Diabetes	81 (15.5%)
Heart failure	13 (2.5%)
With beta blocker therapy	9 (1.7%)
Without beta blocker therapy	4 (0.8%)
Hypertension	265 (50.9%)
Obesity	51 (9.8%)
Renal disease	60 (11.5%)
Use of Selected Drugs or Procedures	
Diuretics	101 (19.4%)
Dressings for venous leg ulcers	15 (2.9%)
Anti-inflammatory agents	251 (48.2%)
Lymphoscintigraphy	2 (0.4%)
Cancer Treatment	
Chemotherapy	330 (63.3%)
Radiation	350 (67.2%)
Surgery	241 (46.3%)
Year of Index Date	
2013–2014	127 (24.4%)
2015–2016	189 (36.3%)
2017–2018	205 (39.3%)

### HNC incidence

81,963 patients aged ≥18 years who had first evidence of HNC between April 1, 2013 and March 31, 2019 were identified. Of these, 26,904 were continuously enrolled during the 1-year period preceding and 1-year period following first evidence of HNC and 16,654 received HNC treatment during the 3-month period following their first HNC diagnosis.

### HNC-related lymphedema incidence

Of the 16,654 HNC patients eligible for evaluation, 1,082 (6.5%) with a diagnosis of lymphedema were identified based on the criteria detailed above.

### Baseline characteristics

The patient population of patients with HNC-related lymphedema is primarily comprised of men (76.8%) with an average age of 58.0 years. Most frequently noted comorbidities included hypertension (50.9%), depression (31.1%), diabetes (15.5%), and renal disease (11.5%). Cancer therapy included radiation (67.2%), chemotherapy (63.3%), and surgery (46.3%).

### HNC-related lymphedema treatment incidence

123,236 patients aged ≥18 years who had first evidence of lymphedema between April 1, 2013 and March 31, 2019 were identified. Of these, 61,890 were continuously enrolled during the 1-year period preceding and 1-year period following first evidence of lymphedema. 521 (0.84%) of the patients had evidence of HNC within one year prior their first documented evaluation for lymphedema treatment.

The details of lymphedema treatment are detailed in Table 2. Of the 521 HNC patients evaluated for lymphedema treatment, 417 (80.0%) patients received 1.5 courses of MLD. 71 (13.6%) patients were prescribed compression garments, and 45 (8.6%) patients received an APCD.

**Table 2:** Use of LED treatments among HNC + LED patients

	HNC + LED patients (*N* = 521)
MLD	417 (80.0%)
Compression garments	71 (13.6%)
SPCD	2 (0.4%)
APCD	45 (8.6%)

## Discussion

Only 6.5% of the 16,654 HNC survivors in this cohort were diagnosed with lymphedema. This is just over half of the historically reported low range of 12% and a small fraction of the incidence of 90% reported in contemporary studies [8–12]. Our database only identified 521 HNC survivors who received treatment for lymphedema suggesting marked undertreatment of the disorder. The primary treatment modality for those referred was MLD (80%) with only a small percentage receiving compassion garments (13.6%) and even fewer (8.6%) receiving APCD.

Under recognition, underdiagnosis, and undertreatment of HNL has potentially profound implications at many levels. Like radiation fibrosis syndrome (RFS), lymphedema is a progressive process associated with protein deposition, fibrosis and sclerosis [5, 8, 24]. Unlike RFS, the protein deposition and fibrosis resulting from lymphedema may be mitigated, at least to some degree, by effective lymphedema therapies such as MLD, compression and APCD utilization [25–29]. This raises the possibility that not identifying and addressing HNL may contribute to progression of fibrosis-related issues by superimposing lymphedema-related fibrosis on top of radiation-related fibrosis. Early evidence suggests APCD has positive benefits for lymphedema control, neck pain, dysphagia, and dyspnea [29]. At the population level, addressing lymphedema in HNC survivors may hold the potential for reduced cost of care to payers and reduced burden of care on oncology clinicians.

HNL is understudied, underrecognized, underdiagnosed, and undertreated despite a growing body of evidence that successfully addressing it in HNC survivors may confer a multitude of benefits. Clinicians involved in the care of HNC patients should familiarize themselves with the epidemiology, diagnostic features, and principles of treating HNL. In addition to traditional MLD, newer tools such APCD may facilitate treatment of HNL and mitigate associated sequelae.

## Appendix

This data extract includes all healthcare claims and enrollment information for all patients with any evidence of the following conditions or procedures from January 2012 through December 2019 (the “study period”):

-  ≥ 1 diagnosis code (irrespective of care setting) for lymphedema (ICD-9: 457.0, 457.1, 757.0; ICD-10: I97.2, I89.0, Q82.0);
-  ≥ 1 diagnosis code (irrespective of care setting) for edema (ICD-9: 782.3; ICD-10: R60.x);
-  ≥ 1 procedure code (irrespective of care setting) for pneumatic compressor segmental home model without calibrated gradient pressure (E0651) or pneumatic compressor segmental home model with calibrated gradient pressure (E0652);
-  ≥ 1 diagnosis code (irrespective of care setting) for head/neck cancer (ICD-9: 140.x-149.x, 160.x, 161.x, 162.0, 170.0–170.1, 176.2, 195.0; ICD-10: C00.x-C14.x, C30.x-C46.x, C76.0); or
-  ≥ 1 diagnosis code (irrespective of care setting) for:
  - Venous ulcer/venous insufficiency (ICD-9: 459.81; ICD-10: I87.2)
  - Varicose veins (ICD-9: 454.x; ICD-10: I83.xxx)
  - Ulcer of lower limbs (ICD-9: 707.1x; ICD-10: L97.xxx)
  - Chronic venous hypertension with ulcer/inflammation (ICD-9: 459.3x; ICD-10: I87.3x)
  - Post-thrombotic syndrome (ICD-9: 459.1x; ICD-10: I87.0xx)
  - Iliocaval venous obstruction (ICD-9: 459.2; ICD-10: I87.1)
