# Correction to: Under recognition and treatment of lymphedema in head and neck cancer survivors – a database study

**DOI:** 10.1007/s00520-023-07809-0

**Published:** 2023-05-15

**Authors:** Michael D. Stubblefield, Derek Weycker

**Affiliations:** 1 Kessler Institute for Rehabilitation, 1199 Pleasant Valley Way, West Orange, NJ 07052 USA; 2 Policy Analysis Inc. (PAI), 822 Boylston Street, Suite 206, Chestnut Hill, MA USA

**Correction to: Supportive Care in Cancer (2023) 31:229**

10.1007/s00520-023-07698-3

The article “Under recognition and treatment of lymphedema in head and neck cancer survivors – a database study”, written by Stubblefield, M.D. and Weycker, D., was originally published Online First without Open Access. After publication in volume 31, issue 4, pages 289–293 the author decided to opt for Open Choice and to make the article an Open Access publication. Therefore, the copyright of the article has been changed to © The Author(s) 2023 and the article is forthwith distributed under the terms of the Creative Commons Attribution 4.0 International License, which permits use, sharing, adaptation, distribution and reproduction in any medium or format, as long as you give appropriate credit to the original author(s) and the source, provide a link to the Creative Commons licence, and indicate if changes were made. The images or other third party material in this article are included in the article’s Creative Commons licence, unless indicated otherwise in a credit line to the material. If material is not included in the article’s Creative Commons licence and your intended use is not permitted by statutory regulation or exceeds the permitted use, you will need to obtain permission directly from the copyright holder. To view a copy of this licence, visit http://creativecommons.org/licenses/by/4.0.

The original article is corrected.

